# Plants Assemble Species Specific Bacterial Communities from Common Core Taxa in Three Arcto-Alpine Climate Zones

**DOI:** 10.3389/fmicb.2017.00012

**Published:** 2017-01-24

**Authors:** Manoj Kumar, Günter Brader, Angela Sessitsch, Anita Mäki, Jan D. van Elsas, Riitta Nissinen

**Affiliations:** ^1^Department of Microbial Ecology, University of GroningenGroningen, Netherlands; ^2^Department of Biological and Environmental Science, University of JyväskyläJyväskylä, Finland; ^3^Health and Environment Department, AIT Austrian Institute of TechnologyTulln, Austria

**Keywords:** endophytic bacteria, arcto-alpine plant, biogeographical diversity, core bacteriome, *Oxyria digyna*, *Saxifraga oppositifolia*

## Abstract

Evidence for the pivotal role of plant-associated bacteria to plant health and productivity has accumulated rapidly in the last years. However, key questions related to what drives plant bacteriomes remain unanswered, among which is the impact of climate zones on plant-associated microbiota. This is particularly true for wild plants in arcto-alpine biomes. Here, we hypothesized that the bacterial communities associated with pioneer plants in these regions have major roles in plant health support, and this is reflected in the formation of climate and host plant specific endophytic communities. We thus compared the bacteriomes associated with the native perennial plants *Oxyria digyna* and *Saxifraga oppositifolia* in three arcto-alpine regions (alpine, low Arctic and high Arctic) with those in the corresponding bulk soils. As expected, the bulk soil bacterial communities in the three regions were significantly different. The relative abundances of *Proteobacteria* decreased progressively from the alpine to the high-arctic soils, whereas those of *Actinobacteria* increased. The candidate division *AD3* and *Acidobacteria* abounded in the low Arctic soils. Furthermore, plant species and geographic region were the major determinants of the structures of the endophere communities. The plants in the alpine region had higher relative abundances of *Proteobacteria*, while plants from the low- and high-arctic regions were dominated by *Firmicutes*. A highly-conserved shared set of ubiquitous bacterial taxa (core bacteriome) was found to occur in the two plant species. *Burkholderiales, Actinomycetales* and *Rhizobiales* were the main taxa in this core, and they were also the main contributors to the differences in the endosphere bacterial community structures across compartments as well as regions. We postulate that the composition of this core is driven by selection by the two plants.

## Introduction

Among the terrestrial environments on Earth, arctic and alpine ecosystems cover about 8% of the global land area, which is more than the area covered by tropical forests (Chapin and Körner, 1996). These arctic and alpine ecosystems have the least biologically usable heat and the lowest diversity of plants (Billings and Mooney, 1968). The plants in these systems are well adapted to cold and short growing seasons and low-nutrient soils. The typical plant species occurring in both biomes are collectively referred to as arcto-alpine vegetation. These plants are important in these soils, as they constitute the prime settlers that are at the basis of the local living ecosystem. It has been hypothesized that the local microbiota plays an important role in the ecological success of these pioneering plants (Borin et al., 2010; Mapelli et al., 2011). While arctic and alpine biomes share many characteristics, including short and cool growing seasons, cold winters and soils with low levels of nutrients, there are also distinct differences: the alpine biome is characterized by high annual and diurnal temperature fluctuation and high solar intensity in the summer and, in general, well-drained soils. The vegetation in the Arctic, on the other hand, experiences weeks to months long polar night in winter and 24-h daylight during the growing season. Moreover, arctic soils are typically water-logged due to underlying permafrost (Körner, 2003). These differences have led to “climatic ecotypes” within arcto-alpine vegetation, where the growth morphology and phenology of the same plant species in different biomes reflect adaptation to distinct climates.

Endophytic bacteria are ubiquitous across both cultivated and wild plants. They have been shown to contribute to major aspects of plant life, including regulation of growth and development, nutrient acquisition and protection from biotic and abiotic stressors (reviewed in Hardoim et al., 2015). Studies conducted mainly in agricultural or model plant systems have offered a rapidly growing insight into the assembly, structure and function of the endophytic communities in plants (Zhang et al., 2006; Compant et al., 2010). Soil type and plant species and genotype are both known to shape the rhizosphere (Garbeva et al., 2004; Berg and Smalla, 2009) and root endosphere communities (Bulgarelli et al., 2013). Rhizosphere soil is considered to be the main source of endophytes (Bulgarelli et al., 2013), but vertical transmission via seeds has also been reported (Puente et al., 2009; Hardoim et al., 2012). However, the factors governing the plant-associated microbiota of perennial wild plants in the aforementioned arcto-alpine soils may differ from those of model or well-fertilized crop plants. For plants in the low-arctic fell tundra, we have previously shown that plant species, rather than sampling site, determines the structure of the endophytic (Nissinen et al., 2012) and rhizospheric (Kumar et al., 2016) microbial communities.

Most bacterial species are considered to be cosmopolitan, as they have been found across biogeographic regions in habitats like soils, sediments, lakes and the sea (Hanson et al., 2012). Interestingly, the bacterial diversity in arctic soils has been shown not to differ from that of other biomes (Chu et al., 2010). With respect to community structure, endemism per region has been observed for bacteria, with some taxa reportedly being restricted to distinct geographical regions (Cho and Tiedje, 2000; Oakley et al., 2010).

The main goal of this study was to investigate the factors that shape the bacterial communities associated with two plant species in three geographic regions, from the high Arctic to the Alps. Our target plant species, *Oxyria digyna* and *Saxifraga oppositifolia*, are arcto-alpine plant species with wide distribution from the high Arctic to the mid-latitude alpine tundra. Both are typical pioneer species that efficiently colonize low-nutrient tundra soils. *O. digyna* is a member of the Polygonaceae (order Caryophyllales), whereas *S. oppositifolia* belongs to the order Saxifragales, which diverged from other core eudicots 114–124 MYA (Soltis et al., 2000; Wikström et al., 2001). We focused on the root endophytic bacteria, and also examined the bacterial communities in the relevant rhizosphere and bulk soil samples.

We hypothesized that (1) geographic region, related to climate zone, determines the diversity and community structure of the soil bacterial communities in the selected habitats, and (2) plants strongly shape the plant-associated communities, resulting in plant species specificity, regardless of the geographic region. We also hypothesized that (3) part of the plant-associated bacteria are consistently present in their hosts, constituting an endophytic core microbiome.

To achieve our aims, we used community DNA based amplicon sequencing targeting the bacterial 16S rRNA gene region and subsequent analyses.

## Materials and methods

### Sampling locations and study sites

**Figure 1:**
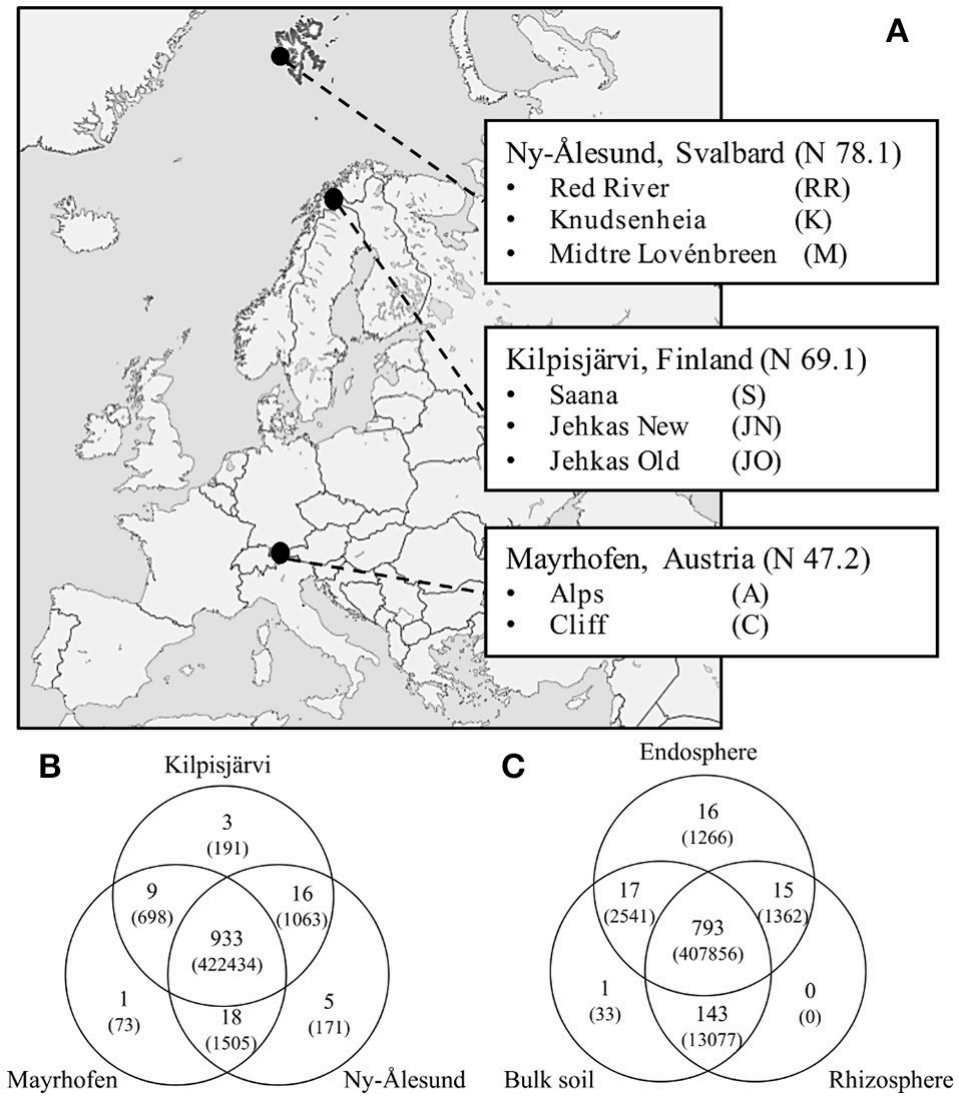
**Sampling sites and OTU distribution. (A)** Map of Europe depicting our three sampling locations Mayrhofen from Austrian Alps, Kilpisjärvi from low-arctic Finnish Lapland and Ny-Ålesund from high-arctic Svalbard archipelago. **(B)** Venn diagrams of shared OTUs (number of reads of respective OTUs) across three regions and **(C)** compartments.

Plant and soil samples were collected from eight sampling sites in three distinct regions representing different climate zones; Ny-Ålesund, high Arctic (3 sampling sites), Kilpisjärvi, low Arctic (3 sampling sites) and Mayrhofen, European Alps (2 sampling sites) (Figure 1A). Kilpisjärvi is at the northwestern Finland and located along the Fenno-Scandinavian border. Its flora is dominated by mountain birch forest in the valleys and by fell tundra at higher elevations. The annual mean temperature is about −2.2°C with plant growth season of ca. 90–100 days. Ny-Ålesund (Svalbard, Norway) is located on an isolated archipelago in the high Arctic; the land cover is dominated by glaciers and permafrost layers, and the mean annual temperature is −4°C. The soil temperatures have been reported to be below zero for more than 250 days per year ranging from −6° to −25°C (Coulson and Hodkinson, 1995). Most biological activity is restricted to less than 10% of the total land mass coupled with about 3 months of plant growing season. The sampling location in the Mayrhofen is located above the tree line south of Mayrhofen over the snow-covered mountains (altitude ca. 2400 m above sea level) in the alpine tundra of the European Alps. Coordinates and details of sampling sites are listed in Supplementary File S1.

### Sample collection and processing

Twelve replicates of bulk soil samples (the top 5 cm soil was removed and soil samples from 5–10 to 10–15 cm, corresponding to major root mass of target plant species, were both used for analysis) and six samples of both *O. digyna* and *S. oppositifolia* (as whole plants with adhering rhizosphere soils) were collected from all sites, except in site “Saana” (Kilpisjärvi) where only *O. digyna* plants were sampled and site “Cliff” (Mayrhofen) where we sampled only 6 bulk soil samples. Sampling was performed during summer 2012. All harvested plants were flowering at the time of the sampling. Rhizosphere and bulk soil samples were processed and stored as specified by Kumar et al. (2016). After removing rhizosphere soils, plant roots were thoroughly washed with water and surface sterilized by immersing the plant material into 3% sodium hypochlorite for 3 min and then subsequently in sterile double distilled water (3 × 90 s). 80–100 mg of root samples were weighed, snap frozen with liquid nitrogen and stored at −80°C for further DNA analysis.

Soil pH and soil organic matter (SOM) content were measured as described in Kumar et al. (2016), while available phosphorous (P) was measured based on Bray No 1 extraction method (Bray and Kurtz, 1945). All the soil chemical analyses were performed in duplicates (2 technical replicates) per sample, and with 4–8 biological replicates per site and sample type (Table 1).

**Table 1:** **Soil physico-chemical properties**.

**Region**	**Sampling site**	**SOM (%)**	**pH**	**Available phosphorous mg/kg**
Mayrhofen	Alps (A) [8]	0.01 (0.002)	7.03 (0.9)	1.84 (1.1)
	Cliff (C) [4]	0.02 (0.002)	4.60 (0.1)	1.48 (0.4)
	Average	0.01 (0.002)	5.81 (0.5)	1.66 (0.8)
Kilpisjärvi	Jehkas New (JN) [8]	0.02 (0.002)	5.55 (0.2)	1.31 (0.4)
	Jehkas Old (JO) [8]	0.02 (0.008)	6.36 (0.4)	0.76 (0.4)
	Saana (S) [8]	0.02 (0.01)	5.49 (0.6)	2.45 (1.5)
	Average	0.02 (0.01)	5.80 (0.5)	1.51 (0.8)
Ny- Ålesund	Knudsenheia (K) [8]	0.03 (0.01)	7.4 (0.9)	0.83 (0.5)
	Midtre Lovénbreen (M) [8]	0.03 (0.03)	6.4 (1.2)	0.63 (0.1)
	Red River (RR) [8]	0.04 (0.01)	7.78 (0.5)	0.34 (0.1)
	Average	0.04 (0.02)	7.20 (0.9)	0.60 (0.3)

*(), Standard deviation values*.

*[], number of biological replicates/sampling site*.

### DNA isolation

Microbial DNA from soil samples were extracted following manufacturer's instruction using MoBio Power soil kit (MoBio, Carlsbad, CA, USA). For soil samples 0.5 g of soil was used instead of 0.25 g because of low microbial counts in our soils (data not shown). For isolation of endophyte samples, Invisorb Spin Plant Mini Kit (STRATEC Biomedical AG, Germany) was used in order to ensure prolonged stability of endophytic DNA in the plant derived samples. Frozen plant tissues were homogenized by bead beating for 45 s with 0.1 mm sterilized glass beads with FastPrep homogenizer (mpbio.com), followed by DNA extraction according to manufacturer's protocol.

### 16S rRNA gene library generation and sequencing

After isolating DNA from all six plant replicates from both plant species, four (rhizo- and endosphere) or eight (bulk soil) samples technically best samples (good DNA yield, good PCR amplification) were included in the next generation sequencing library construction.16S rRNA gene was amplified using primers 799f/1492r (Chelius and Triplett, 2001) and M13-1062f/1390r in a nested approach. The nested primers targeting the V6-V8 regions of 16s rRNA gene enable elimination of plant chloroplast 16S rRNA gene amplicons as well as separation of endophyte amplicons from plant mitochondrial amplicons by size fractionation (799f–1492r, Chelius and Triplett 2001) and produce an amplicon with high phylogenetic coverage and optimal size for IonTorrent sequencing (1062f–1390r). Primers 1062f (Ghyselinck et al., 2013) and 1390r (Zheng et al., 1996) were tagged with M13 sequences to enable sample barcoding as described below and in Mäki et al. (2016). Both reactions had 1 μl of sample DNA, 1x PCR buffer, 1 mg/ml of BSA, 0.2 mM dNTP's, 0.3 μM of each primer and 1250 U/ml GoTaq DNA Polymerase (Promega, WI, USA) in a 30 μl reaction volume. 5–10 and 25–30 ng of soil and endophyte DNA, respectively, was used in the first PCR, and 1 μl of 1:10 diluted amplicons (for bulk and rhizosphere soil samples) and 1 μl of amplicons (for endosphere samples) from the first PCR were used as a template for the second run. Amplifications for both PCR reactions were performed as follows: 3 min denaturation at 95°C followed by 35 cycles of denaturing, annealing, and extension at 95°C for 45 s, 54°C for 45 s, and 72°C for 1 min, respectively. Final extension was carried out at 72°C for 5 min. Prior to library production, the PCR protocol was optimized with regard to several primer pair combinations, PCR protocols and test of PCR blockers to minimize the strong interference of mitochondrial rRNA in *O. digyna* and *S. oppositifolia*. The above described protocol, using high coverage, minimal bias primer pairs, was shown to produce enough eubacterial (endophytic) amplicons with no observable decline in diversity (as detected by T-RFLP) for sequencing, while most alternatives lead to very low amplification levels endophytes and strong mitochondrial signal.

Sequence libraries were prepared by running a third PCR to attach the M-13 barcode system developed by Mäki et al. (2016). Amplicons from second PCR were diluted 1:5 and re-amplified using barcode attached M13 system as forward primer and 1390r-P1 with adaptor A as a reverse primer. PCR mix and conditions were similar as described above, with an exception of using 8 cycles for amplification. Amplified libraries were purified using Agencourt AMPure XP PCR purification system (Beckman Coulter, CA, USA). Purified samples were quantified with Qubit Fluorometer (Invitrogen, MA, USA) and an equivalent DNA quantity of each sample was pooled together. The pooled samples were then size fractionated (size selection range of 350–550 bp) using Pippin Prep (Sage Science, MA, USA) 2% Agarose gel cassette (Marker B) following the manufacturer's protocol. Size fractioned libraries were sequenced using Ion 314 chip kit V2 BC on Ion Torrent PGM (Life Technologies, CA, USA) in Biocenter Oulu, Finland.

### Bioinformatics and statistical analysis

The raw sequence reads were processed using QIIME (Caporaso et al., 2010) and UPARSE (Edgar, 2013) based on a 16S rRNA gene data analysis pipeline developed by Pylro et al. (2014) with slight modifications in quality filtering. Sequences were trimmed by removing sequences with low quality reads (Q score <25) and shorter base pair (<150) length. Furthermore, all the raw reads were trimmed (200 bp), aligned and clustered at 97% identity using USEARCH algorithm (Edgar, 2010). UCLUST algorithm along with Greengenes database (DeSantis et al., 2006) was used to assign taxonomies at 97% identity to the individual OTUs. In total, 426,135 high-quality reads (1468 reads-5331 reads per sample) were clustered into 985 OTUs. For alpha diversity analysis all the samples were rarefied (subsampled) to 1400 reads per sample. Shannon index and species richness were obtained using Univariate Diversity Indices (DIVERSE, PRIMER 6 (PRIMER-E Ltd.)). The differences in diversity indexes between the soil samples and their correlation with soil physico-chemical properties were determined using two-way ANOVA and Pearson correlation (SPSS Statistics, IBM). The significance of the differences between the soil samples were tested by Games-Howell *post-hoc* tests (two-way ANOVA).

To normalize the data for community structure and other analyses all the samples with more than the median reads were rarefied to the median (2780 reads), while the samples with less reads were used as such, as described in Cárcer et al. (2011). In addition, all the singletons and OTUs with less than 50 reads were removed before processing. The influence of sampling site, geographic region, plant compartment, and plant species on bacterial community structures, based on Bray-Curtis distance matrixes of square root transformed abundance data, were analyzed using permutational multivariate analysis of variance (PERMANOVA) and visualized by PCoA ordinations at the OTU level. Taxonomic groups (phyla or OTU) with strongest impact on significant differences between community structures were identified with SIMPER (Similarity Percentages–species contributions), all performed with PRIMER 6 software package with PERMANOVA+ add-on (primer-e.com).

All the Ternary plots were made by calculating the mean relative abundances of OTUs per geographic region/compartment and with the function “ternaryplot” “vcd” (Meyer et al., 2015) from the R package. All other graphs (bar and scatter plots), also based on the mean relative abundances of taxa, were constructed using the R package “graphics.”

### Picking endosphere core OTUs

The highly conserved OTUs (core OTUs) were manually picked by selecting OTUs that were constantly observed (present in at least 3 out of 4 replicates per site) in the endosphere of either *O. digyna* and *S. oppositifolia* or both. To determine the distribution of core OTUs reads across different compartments, the averaged read count per compartment was calculated for each OTU, the averages were summed up and presented as the relative distribution of each of the 13 core OTUs per compartment.

## Results

A total of 426,135 quality-filtered sequence reads was retrieved from the total of 174 samples in our sample set, representing the endospheres and rhizospheres of the two plant species and the corresponding bulk soils from the three geographic regions (Figure 1A). These sequences were separated into 985 OTUs (defined at the 97% cut-off level) and subjected to downstream analyses. Of these, 933 OTUs were present in at least one of the samples from each of the three regions, 43 in two regions and nine were restricted to one region only (Figure 1B). 778 of the 985 OTUs were found in all compartments (bulk soil, rhizosphere soil or endosphere), 190 in two compartments and 17 were compartment-specific (Figure 1C).

### Soil characteristics are different across three arcto-alpine regions

Table 1 lists the soil characteristics in the three geographic regions: Mayrhofen (alpine), Kilpisjärvi (low-arctic) and Ny-Ålesund (high-arctic) (Figure 1A). The Ny-Ålesund [bulk] soils had significantly higher pH (two-way ANOVA, *p* < 0.05) and soil organic matter (SOM) values (two-way ANOVA, *p* < 0.01), and significantly lower levels of available phosphorus (two-way ANOVA, *p* < 0.05) than the Kilpisjärvi and Mayrhofen soils. The Kilpisjärvi soils had the lowest average pH values, but there were no significant differences in the other physico-chemical properties between the Kilpisjärvi and Mayrhofen bulk soils.

### Geographical region and soil properties impact the diversity of the bulk soil, but not of the rhizosphere or endosphere bacterial communities

**Figure 2:**
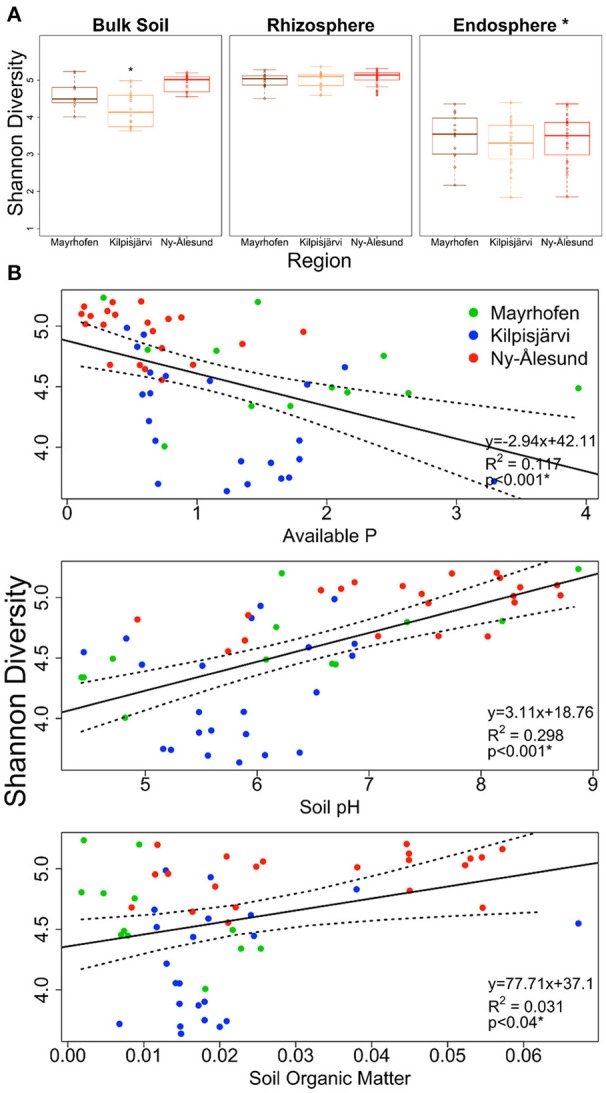
**Estimated Shannon diversity (A)** in bulk soil, rhizosphere soil and endophytic bacterial communities from three climatic regions Mayrhofen, Kilpisjärvi and Ny-Ålesund. **(B)** Scatter plots of bulk soil communities explaining the correlation (Pearson correlation) of Shannon diversity with soil-physico chemical properties from three climatic regions.

The species richness (SR) and α-diversity (Shannon index, SI) values of the bulk soil bacterial communities differed between the geographic regions. The Kilpisjärvi bulk soils (*SR* = 33.95, *SI* = 4.21) had significantly lower richness (two-way ANOVA, *p* < 0.01) and diversity (two-way ANOVA, *p* < 0.01) values than the Mayrhofen (*SR* = 41.04, *SI* = 4.61) and Ny-Ålesund bulk soils (*SR* = 42.55, *SI* = 4.92; Figure 2A). In contrast, there were no significant differences in the diversity levels of the rhizosphere soil or endosphere samples between the regions (Figure 2A).

There was a significant positive relationship of both SR and SI with soil pH (Pearson correlation [2-tailed], SR, *p* < 0.001, SI, *p* < 0.001) and a negative one with the levels of available phosphorus (P) (Pearson correlation [2-tailed], SR, *p* < 0.014, SI, *p* < 0.001). There was a significant positive correlation between SOM and SI, but not between SOM and SR (Figure 2B).

### Bacterial community structures in samples from different regions differ at the phylum level

Collectively, the OTUs from all our samples fell into 21 bacterial phyla. Eight of these, i.e., *Proteobacteria, Actinobacteria, Acidobacteria*, candidate division *AD3, Bacteroidetes, Firmicutes, Chloroflexi* and *Gemmatimonadetes*, were prominent, collectively making up about 97% of the total microbiome. The remaining 13 phyla were present at less than 1% relative abundance each.

**Figure 3:**
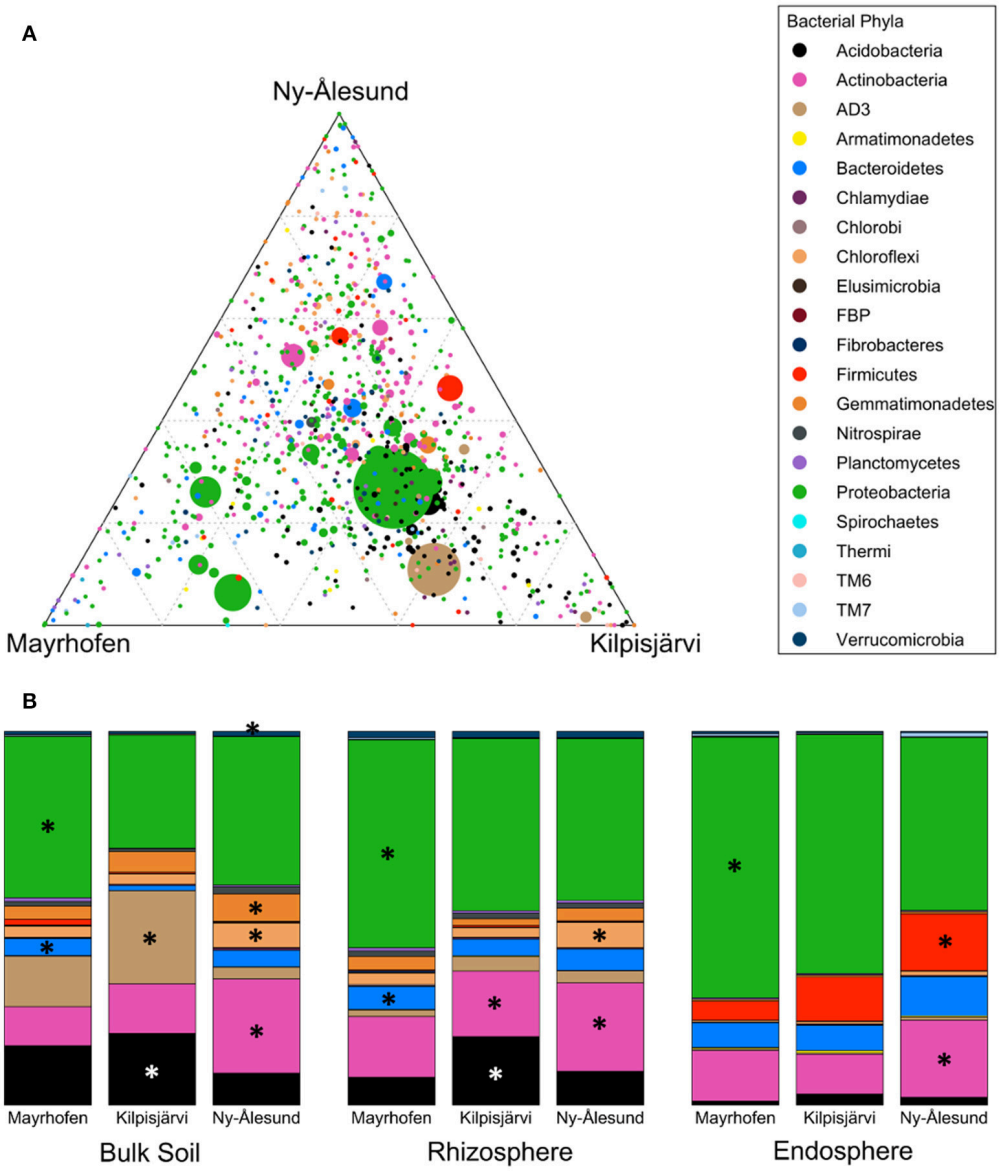
**Distribution of OTUs and phyla across regions (A)** Ternary plot of OTU distribution across three climatic regions. Each circle represents one OTU, and the size, color and position of the circle represent its relative abundance, bacterial phylum and affiliation of the OTU with the different regions, respectively. **(B)** Average relative abundances of bacterial phyla distributed across different regions in Bulk soil samples, Rhizosphere soil samples and Endosphere samples. Major phyla (average relative abundance above 1%) with significantly differential distribution (as detected by Kruskal-Wallis analysis) are marked with asteriskses.

Bacterial community structures in the samples from the different regions were significantly different at phylum level (PERMANOVA *F* = 8.1155, *p* = 0.001). SIMPER analyses confirmed that *Proteobacteria, Acidobacteria, AD3* and *Actinobacteria* were the main phyla contributing to the overall dissimilarities between the regions (Table 2). *Proteobacteria* were relatively more abundant in the alpine (Mayrhofen; average relative abundance 57%) than in the arctic regions (46% in Kilpisjärvi and 43% in Ny-Ålesund). The phylum *Acidobacteria* and the candidate division *AD3* were observed in higher average relative abundances in Kilpisjärvi than in the other two regions (Figures 3A,B). The *AD3* candidate division had reduced diversity (Figure 3A), with a single abundant OTU (OTU 10) dominating the Kilpisjärvi bulk soil samples, representing about 25% of the total bulk soil community. The Ny-Ålesund samples were enriched with *Actinobacteria* (Figure 3A), with average relative abundances in Kilpisjärvi = 14%, Mayrhofen = 14% and Ny-Ålesund = 23%.

**Table 2:** **Contributions of variables to similarity (SIMPER) analysis based on Bray-Curtis dissimilarity indexes at phylum level identifying the major phyla driving the dissimilarities between different regions or compartments**.

**Phylum**	**Average abundance**	**Average abundance**	**Contribution to dissimilarity %**
**Bacterial phylum level–regions (Pseudo-F: 8.116 p: 0.001)**			
**t: 2.087, p: 0.016**	**Mayrhofen**	**Kilpisjärvi**	
*Proteobacteria*	56.93	46.14	27.77
*Acidobacteria*	7.68	13.74	17.35
*AD3*	4.48	10.04	15.66
*Actinobacteria*	13.64	13.88	13.36
*Firmicutes*	2.36	4.06	7.17
*Bacteroidetes*	5.82	4.09	5.72
*Gemmatimonadetes*	2.5	2.69	3.9
**t: 2.578, p: 0.001**	**Mayrhofen**	**Ny-Ålesund**	
*Proteobacteria*	56.93	43.3	25.91
*Actinobacteria*	13.64	23.38	18.14
*Acidobacteria*	7.68	6.18	12.7
*Firmicutes*	2.36	5.48	9.62
*AD3*	4.48	2.23	8.19
*Bacteroidetes*	5.82	6.99	7.48
*Chloroflexi*	2.17	4.83	5.79
*Gemmatimonadetes*	2.5	3.68	5
**t: 3.495, p: 0.001**	**Kilpisjärvi**	**Ny-Ålesund**	
*Proteobacteria*	46.14	43.3	19.47
*Actinobacteria*	13.88	23.38	18.25
*Acidobacteria*	13.74	6.18	16.32
*AD3*	10.04	2.23	13.64
*Firmicutes*	4.06	5.48	10.04
*Bacteroidetes*	4.09	6.99	6.92
*Chloroflexi*	1.99	4.83	5.16
*Gemmatimonadetes*	2.69	3.68	4.52
**Bacterial phylum level–compartment (Pseudo-F: 64.371, p: 0.001)**			
**t: 3.915, p: 0.001**	**Bulk Soil**	**Rhizosphere**	
*AD3*	13.59	2.89	20.44
*Proteobacteria*	36.92	47.47	19.58
*Actinobacteria*	17.7	19.71	18.99
*Acidobacteria*	13.84	11.77	18.92
*Gemmatimonadetes*	5.88	2.88	5.97
*Bacteroidetes*	3.33	5.47	4.78
*Chloroflexi*	4.39	4.38	4.46
**t: 9.353, p: 0.001**	**Bulk Soil**	**Endosphere**	
*Proteobacteria*	36.92	58.06	24.91
*AD3*	13.59	0.55	14.37
*Acidobacteria*	13.84	2.16	13.47
*Actinobacteria*	17.7	15.45	13.32
*Firmicutes*	0.56	11.81	12.16
*Bacteroidetes*	3.33	8.16	6.69
*Gemmatimonadetes*	5.88	0.48	5.8
*Chloroflexi*	4.39	0.76	4.12
**t: 8.569, p: 0.001**	**Rhizosphere**	**Endosphere**	
*Proteobacteria*	47.47	58.06	23.81
*Firmicutes*	0.29	11.81	16.5
*Acidobacteria*	11.77	2.16	15.09
*Actinobacteria*	19.71	15.45	14.71
*Bacteroidetes*	5.47	8.16	8.24
*Chloroflexi*	4.38	0.76	5.51
*AD3*	2.89	0.55	4.44
*Gemmatimonadetes*	2.88	0.48	3.52

*Pairwise Permutational multivariate analyses (PERMANOVA) were performed prior to SIMPER to test for significant differences between the tested groups. Pseudo-F and p-values, or t and p- values from PERMANOVA are given for each factor and group pair, respectively*.

The increased relative abundances of *Proteobacteria* in Mayrhofen, *Acidobacteria* and *AD3* in Kilpisjärvi and *Actinobacteria* in Ny-Ålesund were also consistent in the communities in the different compartments (bulk soil, rhizosphere soil, endosphere) (Supplementary File S2), with the exception of *AD3*, which was present at very low abundances in the endosphere (<0.9%) in all three regions (Table 2). Additionally, the relative abundances of *Firmicutes* in the endosphere samples increased with increasing latitude, being lowest in Mayrhofen and highest in Ny-Ålesund.

### *Firmicutes, Proteobacteria*, and *Bacteroidetes* dominate endosphere communities

**Figure 4:**
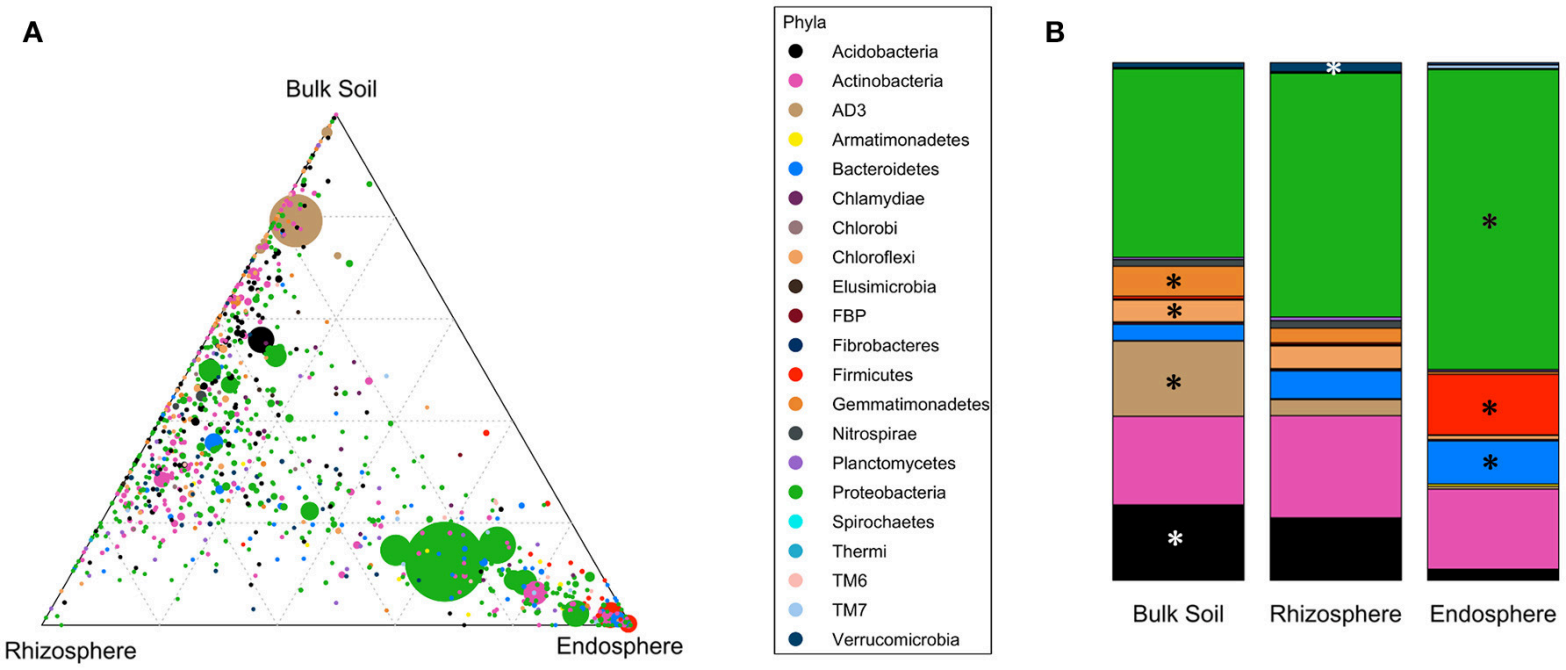
**Distribution of OTUs and phyla across different compartments. (A)** Ternary plot of all OTUs plotted based on the compartment specificity. Each circle represents one OTU. The size, color and position of each OTU represents it relative abundance, bacterial phyla and affiliation of the OTU with different compartments, respectively. **(B)** Distribution of average relative abundance of selected major bacterial phyla from all three regions across the compartments. Major phyla (average relative abundance above 1%) with significantly differential distribution (detected by Kruskal-Wallis analysis) are marked with asteriskses.

Bacterial community structures were clearly different in the different compartments at the phylum level (PERMANOVA pseudo-F = 64.371, *p* = 0.001; Table 2). These differences were mainly driven by strong relative enrichment of *Firmicutes* in the endosphere-derived sequence data sets, compared to their very low abundances in the bulk and rhizosphere soils (Figure 4, Table 2). The relative abundances of *Proteobacteria* and *Bacteroidetes* increased progressively from bulk to rhizosphere soil to the endosphere, with a concomitant decrease in those of candidate division *AD3, Gemmatimonadetes* and *Chloroflexi*, which collectively constituted <4% of endosphere communities (Table 2, Figure 4B). This trend was similar in all three geographic regions.

The divergence of the endosphere communities from the soil communities was also evident at the class level. For example, OTUs representing the actinobacterial class *Thermoleophilia* were abundant in the bulk and rhizosphere soil communities, whereas these were rare in the endosphere communities. The latter were dominated by the class *Actinobacteria* (Class, order and family level analyses in Supplementary File S2).

### Compartment impacts bacterial diversity and community structures more than geographic region or sampling site

The diversity values of the endosphere communities, analyzed at the OTU level, were significantly lower than those of the bulk or rhizosphere soil communities (Figure 2A). The rhizosphere soils had the highest diversity values, but the differences between rhizosphere and bulk soils were not significant (two-way ANOVA, *p* > 0.05, Figure 2A). However, we observed no such differences between the plant species, as *O. digyna* and *S. oppositifolia* had similar SR and SI indices in the rhizo- as well as the endosphere communities.

**Figure 5:**
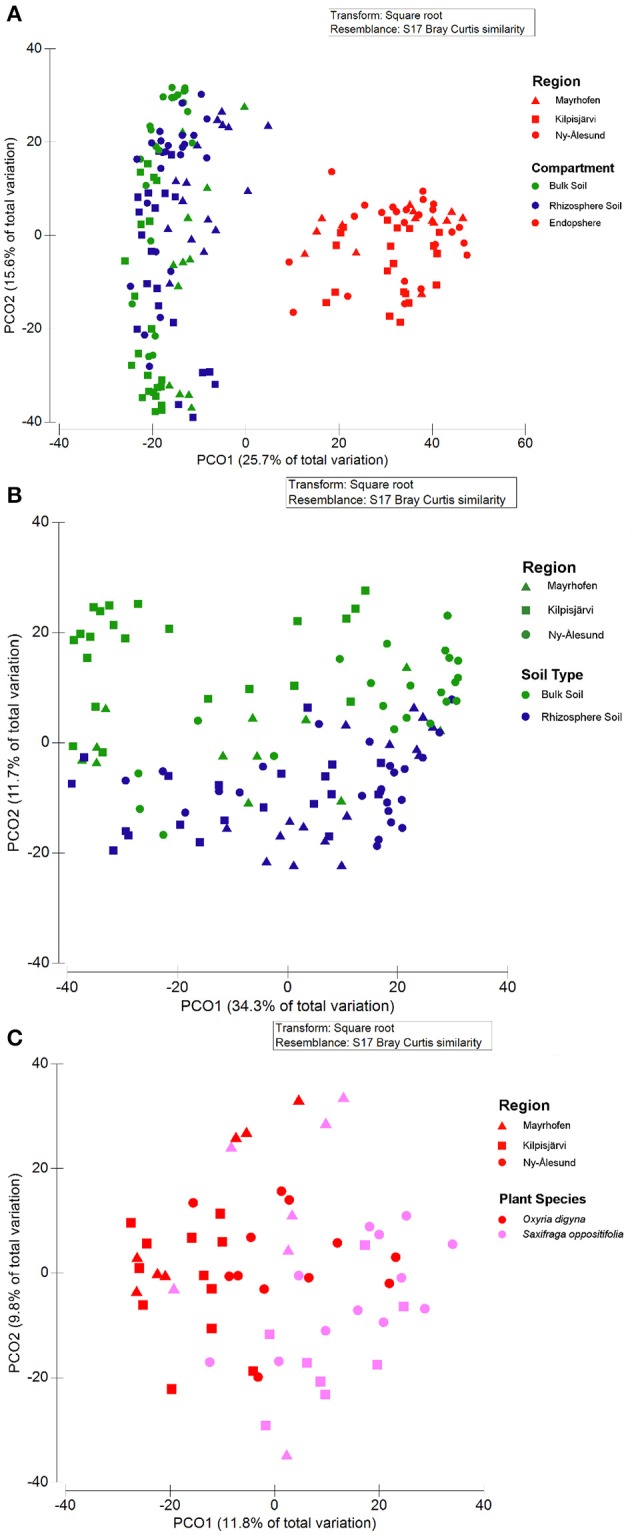
**Principal Coordinate Analysis (PCoA) plots of bacterial communities from bulk soils, rhizosphere soils and endospheres of *O. digyna* and *S. oppositifolia* from three climatic regions Mayrhofen, Kilpisjärvi and Ny-Ålesund. (A)** All samples, **(B)** Bulk soils and rhizosphere soils, **(C)** Endospheres from *O. digyna* and *S. oppositifolia*. The symbol colors correspond to compartment **(A,B)** or plant species **(C)** and the shapes of the symbols correspond to the geographic regions. Compartment, region and plant species all had significant impact on community structures in global as well as in pair wise analyses (PERMANOVA *p* = 0.001). All ordinations are based on Bray-Curtis distance matrixes.

Also, the community structures of the endosphere bacterial communities differed clearly from those of the bulk and rhizosphere soil ones across all three regions, as demonstrated by PCoA (Figure 5A). A separate analysis of the soil-derived samples revealed that the bulk soil communities diverged from the rhizosphere soil ones in the three regions (Figure 5B). This was supported by PERMANOVA, where compartment was identified as a significant and strong driver of the differences between bacterial community structures (Pseudo-F = 30.962, *p* < 0.001, Table 3). Pair-wise analyses of the community structures supported the PCoA analyses, with significant (*p* = 0.001) differences between the endosphere and bulk soil, endosphere and rhizosphere and rhizosphere and bulk soil communities, and *t*-values of 6.339, 6.39, and 3.134, respectively.

**Table 3:** **Permutational multivariate analysis (PERMANOVA) of factors impacting differences between community structures of bacteria at OUT level from different climatic regions, sampling sites, compartments, or plant species**.

**Factor**	**df**	**SS**	**MS**	**Pseudo-F**	***p*****-value(perm)**
**PERMANOVA, SINGLE FACTOR ANALYSIS**					
**All Samples**					
Compartment	2	1.02E + 05	51,132	30.96	0.001
Site	7	56,283	8040	4.064	0.001
Region	2	27,369	13,685	6.549	0.001
**Bulk Soil**					
Site	7	43,653	6236	7.671	0.001
Region	2	21,221	10,610	9.150	0.001
**Rhizosphere Soil**					
Site	7	28,835	4119	5.073	0.001
Region	2	12,352	6176	5.996	0.001
**Endosphere**					
Site	7	29,679	4239	2.142	0.001
Region	2	11,842	5921	2.788	0.001
**PERMANOVA, TWO-FACTOR ANALYSIS**					
**Rhizosphere Soil**					
Region	2	11,466	5732	5.886	0.001
Plant species	1	2910	2910	2.988	0.007
Region × Plant species	2	2983	1491	1.531	0.067
Residuals	54	52,599	974		
**Endosphere**					
Region	2	11,659	5829	2.968	0.001
Plant species	1	7922	7922	4.033	0.001
Region × Plant species	2	6383	3191	1.625	0.003
Residuals	52	1.02E + 05	1964		

In addition to compartment, sampling site (pseudo-F = 4.0646, *p* < 0.001) and region (Pseudo-F = 6.5495, *p* < 0.001) both had significant effects on the bacterial community structures, although these factors had less impact than compartment (Table 3). PERMANOVA performed on each of the compartments separately revealed that region and sampling site had the greatest influence on the structure of bulk soil communities (Pseudo-F = 9.1503, *p* < 0.001 and Pseudo-F = 7.6707, *p* < 0.001, respectively) with their influence decreasing for the rhizosphere (Pseudo-F = 5.9962, *p* ≤ 0.001 and Pseudo-F = 5.0728, *p* ≤ 0.001, respectively) and endosphere (Pseudo-F = 2.7877, *p* ≤ 0.001 and Pseudo-F = 2.1418, *p* < 0.001, respectively; Table 3). Interestingly, region shaped community structures more than sampling site for all compartments, indicating an impact of bioclimatic conditions (Table 3). Thus, in further analyses, we focused on comparing communities from the different regions and plant species.

### Plant species and region both impact endosphere bacterial community structures

PERMANOVA identified both plant species and geographic region as significant drivers of the community structures of the rhizosphere soil communities (PERMANOVA *p* < 0.01), but region (Pseudo-F = 5.8857) had more impact on the differences than plant species (Pseudo-F = 2.9879; Table 3). In contrast, while plant species, region and their interaction all had significant impact on endosphere community structures (PERMANOVA, *p* < 0.01), plant species had stronger impact on the differences between the communities (Pseudo-F = 4.0332) than region or interaction between these factors (Pseudo-F = 2.9678 and Pseudo-F = 1.6249, respectively; Table 3). The endosphere communities from all three regions, being relatively similar to each other, tended to diverge based on plant species (*O. digyna* or *S. oppositifolia*) on the first two axes in the PCoA ordination (Figure 5C), while we did not observe plant species specific clustering in the PCoA of the corresponding rhizosphere communities (data not shown).

On the basis of the above analyses, we found partial support for our hypothesis that plant species strongly shape the plant-associated bacterial communities, as this factor emerged as the major (albeit not the only) significant driver of the endosphere bacterial community structures over multiple sites and several regions (climate zones). Plant species also had a small, but significant impact on the rhizosphere community structures, but these were mainly determined by geographic factors.

### Differences in the endosphere bacterial community structures between the two plant species are explained by differential acquisition of shared bacterial taxa

**Figure 6:**
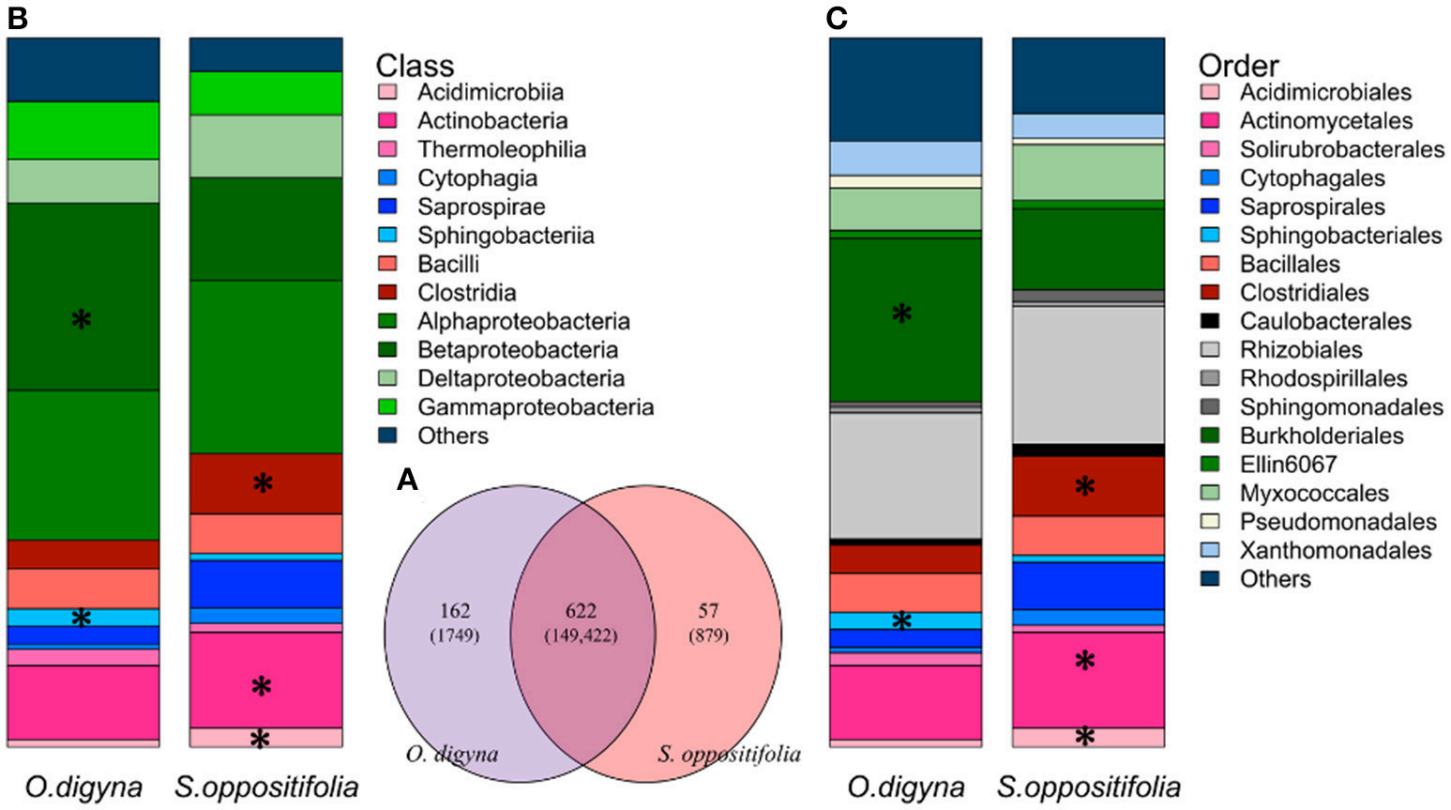
**(A)** Venn diagram of shared and plant species specific OTUs (number of reads of the respective OTUs) between *O. digyna* and *S. oppositifolia* from all the endosphere samples. **(B,C)** Average relative abundance of endophytic bacterial communities associated with *O. digyna* and *S. oppositifolia* endosphere samples at different taxonomical level: **(B)** bacterial class, **(C)** bacterial order. Only major bacterial orders and classes are shown. Major bacterial classes or orders (with average relative abundance above 1% in endosphere) with significantly different distribution (detected by Kruskal-Wallis analysis) are marked with asteriskses.

Remarkably, the majority of the endosphere bacterial taxa was present in both plant species, but in different relative abundances. A total of 841 OTUs was found in the endosphere samples, comprising 152,050 reads. A vast majority, i.e., 612 OTUs (149,422 reads, 98.3% of all endosphere reads), was shared between the two plant species (Figure 6A), and many of these OTUs were consistently enriched along plant species. For example, OTUs representing *Sphingobacteriales* (*Sphingobacteriia, Bacteroidetes*), *Burkholderiales* (β-proteobacteria) and *Bradyrhizobiaceae* were enriched in the *O. digyna* samples, while OTUs in the *Clostridiales*, along with *Actinobacteria*, and *Acidimicrobiia* as well as several OTUs representing *Myxococcales* and *Saprospirales* were relatively more abundant in *S. oppositifolia* across the three climate zones (Figures 6B,C, Supplementary File S2). These were also identified as the main OTUs responsible for plant species specific community structures in the SIMPER analysis (Table 4). In addition to the shared bacterial taxa, 162 OTUs (1749 reads, 1.1% of the total endosphere reads) and 57 OTUs (879 reads, 0.6%) were observed only in *O. digyna* and *S. oppositifolia*, respectively (Figure 6A).

**Table 4:** **Twenty key OTUs shaping the endosphere communities in *O. digyna* and *S. oppositifolia* in the three regions identified by SIMPER (Contributions of variables to similarity analysis)**.

**OTU #**	**Endo vs. Rhizo**	**Plant species**	**Region**	**Phyla**	**Class**	**Order**	**Family**	**Genus**	**Species**
OTU_21	0.97^*^	1.71^*^	1.69^*^	*Firmicutes*	*Clostridia*	*Clostridiales*	*Clostridiaceae*	*Clostridium*	
**OTU_5**	0.95^*^	1.01^*^	1.04^*^	*Firmicutes*	*Bacilli*	*Bacillales*	*Planococcaceae*		
**OTU_2**	0.93^*^	1.73^*^	1.87^*^	*Proteobacteria*	*α-proteobacteria*	*Rhizobiales*	*Bradyrhizobiaceae*	*Bradyrhizobium*	
OTU_3	0.81^*^	1.59^*^	1.68^*^	*Proteobacteria*	*δ-proteobacteria*	*Myxococcales*	*Haliangiaceae*		
**OTU_15**	0.73^*^	1.28^*^	1.20^*^	*Actinobacteria*	*Actinobacteria*	*Actinomycetales*	*Micrococcaceae*	*Kocuria*	
**OTU_8**	0.71^*^	1.52^*^	1.79^*^	*Proteobacteria*	*β-proteobacteria*	*Burkholderiales*	*Oxalobacteraceae*	*Janthinobacterium*	*lividum*
**OTU_33**	0.65^*^	0.99^*^	0.97^*^	*Actinobacteria*	*Actinobacteria*	*Actinomycetales*	*Micromonosporaceae*		
**OTU_4**	0.62^*^	1.43^*^	1.24^*^	*Proteobacteria*	*β -proteobacteria*	*Burkholderiales*	*Comamonadaceae*		
OTU_706	0.62^*^	1.43^*^	1.25^*^	*Bacteroidetes*	*Saprospirae*	*Saprospirales*	*Chitinophagaceae*		
**OTU_13**	0.53^*^	1.12^*^	1.24^*^	*Proteobacteria*	*β -proteobacteria*	*Burkholderiales*	*Comamonadaceae*	*Methylibium*	
OTU_84	0.5^*^	0.99^*^	1.17^*^	*Proteobacteria*	*β -proteobacteria*	*Burkholderiales*	*Comamonadaceae*	*Limnohabitans*	
OTU_48	0.4	0.69^*^	0.78^*^	*Proteobacteria*	*γ-proteobacteria*	*Pseudomonadales*	*Pseudomonadaceae*	*Pseudomonas*	
**OTU_16**	0.37	0.65^*^	0.63	*Proteobacteria*	*α -proteobacteria*	*Rhizobiales*	*Hyphomicrobiaceae*	*Rhodoplanes*	
OTU_22	0.37	0.74^*^	0.79^*^	*Proteobacteria*	*β -proteobacteria*	*Burkholderiales*	*Comamonadaceae*		
OTU_36	0.37	0.92^*^	0.72^*^	*Actinobacteria*	*Actinobacteria*	*Actinomycetales*			
OTU_23	0.29	0.71^*^	0.59	*Proteobacteria*	*γ -proteobacteria*	*Xanthomonadales*	*Sinobacteraceae*	*Steroidobacter*	
OTU_26	0.28	0.64^*^	0.62	*Proteobacteria*	*Un_Proteobacteria*				
OTU_41	0.27	0.56^*^	0.51	*Proteobacteria*	*γ -proteobacteria*	*Xanthomonadales*	*Sinobacteraceae*		
OTU_37	0.26	0.71^*^	0.54	*Proteobacteria*	*α -proteobacteria*	*Rhizobiales*	*Hyphomicrobiaceae*		
OTU_83	0.26	0.59^*^	0.47	*Actinobacteria*	*Acidimicrobiia*	*Acidimicrobiales*			

*Numerical values indicate % contribution of the respective OTUs in determining the difference in community composition between endosphere and rhizosphere, between O. digyna and S. oppositifolia and between the three regions*.

* *indicate the top 20 OTUs strongly contributing to the differences in community structures between the compartments, plant species and geographic regions. OTUs which are also part of tightly associated OTUs were highlighted by bold letters in the OTU # column*.

### Thirteen bacterial taxa are highly conserved in the *O. digyna* and *S. oppositifolia* endosphere communities in all three regions, constituting a major portion of these

**Figure 7:**
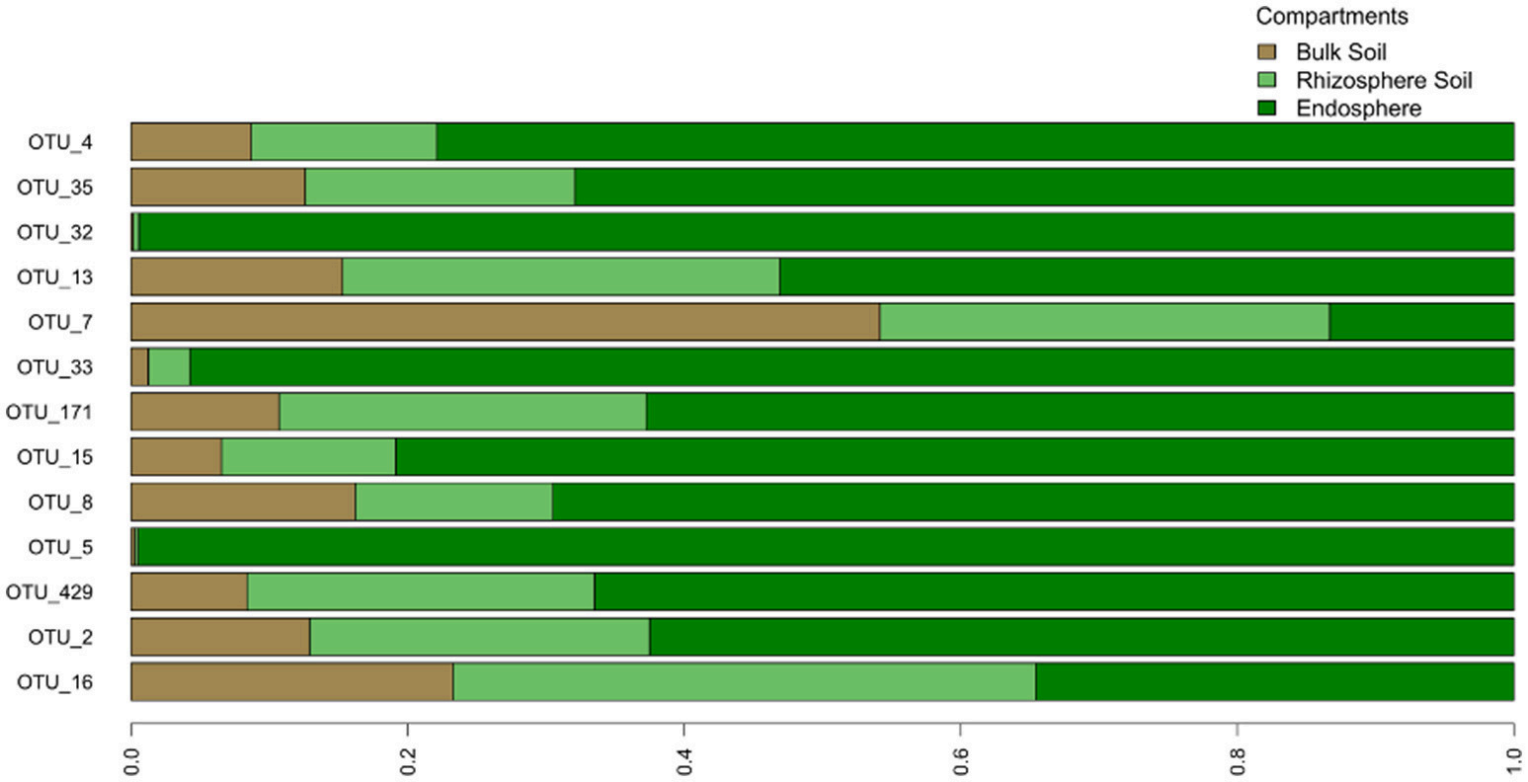
**Relative distribution of core OTUs' reads across different compartments**. The graph is based on average read count of each OTU in different compartments.

We examined the bacterial taxa that were highly conserved (belonging to the “tight” core) in the *O. digyna* or *S. oppositifolia* endospheres using as a criterion “OTUs present in at least three out of four endosphere samples per plant species across all sampling sites and regions.” Thirteen such OTUs were found, of which five, representing *Bradyrhizobium* (2 OTUs), *Rhodoplanes* (α*-Proteobacteria*), *Janthinobacterium* (β*-Proteobacteria*) and *Planococcaceae* (*Firmicutes*), were consistently present in both plant species (Table 5). Additionally, eight OTUs were consistently present in just one of the plant species. Thus *O. digyna* specific core OTUs belonged to *Comamonadaceae* (β*-Proteobacteria*) and *Enterobacteriaceae* (γ*-Proteobacteria*), whereas *S. oppositifolia* specific core OTUs belonged to *Micromonosporaceae, Micrococcaceae* (*Actinobacteria*), *Bradyrhizobiaceae* (α*-Proteobacteria*) and unidentified β*-Proteobacteria* (Table 5). Collectively, these (highly conserved) core OTUs accounted for 38% of the total reads in the endosphere communities. Significantly, eleven of these core OTUs (all except OTUs 171 and 429, Table 5) were among the main drivers of the divergence of the endosphere communities of the two plant species (Table 4). They also explained the differences between the endosphere and the soil bacterial communities, and those between the endosphere communities in the different geographical regions (Table 4). Of the 13 core OTUs, 11 were predominantly present in the plant associated compartments, as over 75% of their reads were detected in the endosphere, and over 80% in the endo- or rhizosphere (Figure 7).

**Table 5:** **Highly conserved core OTUs of *O. digyna* and *S. oppositifolia* endospheres**.

**OTU #**	**Phyla**	**Class**	**Order**	**Family**	**Genus**	**Species**
**CORE OTUS OF BOTH PLANT SPECIES**						
OTU_16	*Proteobacteria*	*α-proteobacteria*	*Rhizobiales*	*Hyphomicrobiaceae*	*Rhodoplanes*	
OTU_2	*Proteobacteria*	*α-proteobacteria*	*Rhizobiales*	*Bradyrhizobiaceae*	*Bradyrhizobium*	
OTU_429	*Proteobacteria*	*α-proteobacteria*	*Rhizobiales*	*Bradyrhizobiaceae*		
OTU_5	*Firmicutes*	*Bacilli*	*Bacillales*	*Planococcaceae*		
OTU_8	*Proteobacteria*	*β-proteobacteria*	*Burkholderiales*	*Oxalobacteraceae*	*Janthinobacterium*	*lividum*
**ADDITIONAL CORE OTUS OF *S. OPPOSITIFOLIA***						
OTU_15	*Actinobacteria*	*Actinobacteria*	*Actinomycetales*	*Micrococcaceae*	*Kocuria*	
OTU_171	*Proteobacteria*	*α -proteobacteria*	*Rhizobiales*	*Bradyrhizobiaceae*		
OTU_33	*Actinobacteria*	*Actinobacteria*	*Actinomycetales*	*Micromonosporaceae*		
OTU_7	*Proteobacteria*	*β -proteobacteria*	*Ellin6067*	*Un_Ellin6067*		
**ADDITIONAL CORE OTUS OF *O. DIGYNA***						
OTU_13	*Proteobacteria*	*β-proteobacteria*	*Burkholderiales*	*Comamonadaceae*	*Methylibium*	
OTU_32	*Proteobacteria*	*γ-proteobacteria*	*Enterobacteriales*	*Enterobacteriaceae*		
OTU_35	*Proteobacteria*	*β-proteobacteria*	*Burkholderiales*	*Comamonadaceae*		
OTU_4	*Proteobacteria*	*β-proteobacteria*	*Burkholderiales*	*Comamonadaceae*		

*OTUs present in minimum of three endosphere samples out of four in all sampling sites in all regions per plant species are included*.

## Discussion

### Factors shaping the bacterial diversity in soils across three climatic regions

In this study, we examined the bacterial communities in three regions spanning over 3000 km in distance, i.e., Mayrhofen (alpine), Kilpisjärvi (low-arctic) and Ny-Ålesund (high-arctic). In these three regions, the climatic conditions are clearly different. The highest bacterial species richness and diversity values in the bulk soils were found in the Ny-Ålesund samples, which was consistent with data by Chu et al. (2011) and Neufeld and Mohn (2005) who also detected highest bacterial diversities in high northern latitudes. However, our data stand in contrast to those from Yergeau et al. (2007), who reported decreasing bacterial diversities in Antarctic soils with increasing latitude toward the south pole. We found a clear positive correlation of bacterial diversity with soil pH and SOM, and a negative correlation with the level of available P, agreeing with studies that put forth soil pH as a major driver of bacterial diversity (Fierer and Jackson, 2006; Lauber et al., 2009; Rousk et al., 2010; Fierer and Lennon, 2011; Shi et al., 2015). Soil nutritional status and available P have also been shown to significantly impact bacterial diversity (Siciliano et al., 2014).

With respect to compartment, the endosphere bacterial communities were significantly less diverse than those in the corresponding soils. However, in contrast with studies from other soils (Smalla et al., 2001; Kowalchuk et al., 2002; Inceoğlu et al., 2011), where rhizosphere soil communities have been reported to be less diverse and rich than bulk soil ones, we observed a trend toward higher richness and diversity in the rhizosphere than in the corresponding bulk soils, although these differences were not statistically significant. This trend was similar to findings in a previous study from the Kilpisjärvi site, where the rhizosphere samples had highest richness and diversity (Kumar et al., 2016). Miniaci et al. (2007) and Coleman-Derr et al. (2016), studying low-SOM glacier forefield or desert soils, respectively, also observed higher bacterial diversity and richness values in the rhizospheres than in the corresponding bulk soils. Further, Yergeau et al. (2007) found that, although soil bacterial diversities in unvegetated Antarctic fell-field soils decreased with increasing (southern) latitude, those from vegetated sites did not. This suggests that a plant-incited “protective or nutritional” effect on bacterial communities becomes increasingly more important in soils in which conditions are challenging.

### Specific OTUs determine the divergence of the soil bacteriomes across three regions

In this study, we detected only few “endemic” bacterial OTUs, as the great majority of the bacterial taxa was found in all three, geographically distant, regions. However, these taxa were present in very different relative abundances, leading to region-driven community structures. Roughly, proteobacterial taxa decreased and Gram-positive ones increased toward the north, with *Acidobacteria* and candidate division *AD3* being enriched in the Kilpisjärvi samples. This clear progressive change in bacterial community structures hints at specific effects of the shifting local conditions on the aforementioned taxa. Thus, habitat filtering rather than [long-distance] dispersal impacts the bacterial community compositions across the three cold climate sites.

The dominance of *Proteobacteria* in the bulk soil samples from Mayrhofen was consistent with findings by Margesin et al. (2009) in alpine soils. Moreover, corroborating earlier studies (Männistö et al., 2007, 2013), the high abundance of *Acidobacteria* was likely linked to the low pH in the Kilpisjärvi soils (Chu et al., 2010; Griffiths et al., 2011). Also, the high abundance of candidate division *AD3* in Kilpisjärvi (Figure 5B) was consistent with similar findings for the Mitchell peninsula in Antarctica (Ji et al., 2015) and low-nutrient sandy soils (Zhou et al., 2003). However, earlier studies by Männistö et al. (2007, 2013) have not detected candidate division *AD3* in high-SOM Kilpisjärvi soils. We here assume that the candidate division *AD3* members that were found are well adapted to the [low SOM/ low nutrient] soils. Alternatively, their absence from the previous data sets might be due to different 16S rRNA targeting primers used in the different studies.

### Compartment is the primary driver of bacterial community structures

A striking observation was that both *O. digyna* and *S. oppositifolia* sampled in any of the three regions exhibited quite similar endosphere bacterial communities. We previously observed compartmental influence between bulk and rhizosphere soils of *O. digyna* and *S. oppositifolia* (Kumar et al., 2016), and so extended this to the endospheres that were addressed in the current study. Clearly, even though the bulk soil bacterial communities were influenced by region and sampling site, which may relate to soil edaphic factors, the plant endospheres shared similar bacterial endophytes across the three regions. This points to a strong and specific filtering effect of the two pioneering plants that were studied, allowing similar bacteria to colonize plants from the widely divergent soils in different regions.

As a token of the plant-incited filtering effect, members of the *Proteobacteria, Actinobacteria, Bacteroidetes* and *Firmicutes* dominated the endosphere bacterial communities. Several other studies, performed with both agricultural and wild plants, also reported these four taxa to be dominant in several endospheres, with *Proteobacteria* being the most dominant one (Coleman-Derr et al., 2016; Santoyo et al., 2016; Zhao et al., 2016). Other taxa, including *Acidobacteria*, candidate division *AD3, Chloroflexi* and *Gemmatimonadetes*, were virtually absent from the endosphere. A general underrepresentation of *Acidobacteria* in the endosphere has also been observed in other systems (Edwards et al., 2015; Zarraonaindia et al., 2015; Coleman-Derr et al., 2016). The enrichment of *Firmicutes* in the endosphere samples in this study was mainly ascribed to the raised abundance of OTUs belonging to the *Clostridia* (in particular OTU 21; genus *Clostridium)*. Possibly, such organisms might have been selected for their capacities to fix nitrogen in the cold and often water-logged soils (Rosenblueth and Martínez-Romero, 2006) in the permafrost-impacted Arctic sites. This hypothesis is supported by our [unpublished] observations, that *nifH* gene libraries prepared from the same plants as used in the current study are dominated by *Clostridium*-type genes in the (high) Arctic. Although *Clostridium* has been described as a strictly anaerobic genus, members of this genus have been shown to fix nitrogen in rice roots (Minamisawa et al., 2004), and survive in the potato endosphere in aerobic conditions (Shabuer et al., 2015). Interestingly, *nifH* genes of *Clostridium* spp. have been reported to be frequent in soil samples from the Canadian high Arctic (Deslippe and Egger, 2006). Similarly, in our study, plants were sampled in early growing season, when these started flowering and snow was melting in most sampling sites.

### A small set of highly conserved OTUs shapes the endosphere bacterial communities in two arcto-alpine plant species

*O. digyna* and *S. oppositifolia*, the target plant species in this study, are both perennial herbs with similar habitat requirements, producing tap root systems of similar size and depth; the plants often grow at close proximity to each other. However, they are taxonomically quite distant (Soltis et al., 2000; Wikström et al., 2001) and have differing mycorrhizal associations. *O. digyna* is non-mycorrhizal, whereas *S. oppositifolia* is endomycorrhizal, which is likely to have strong impact on its nutrient acquisition efficiency.

Despite these differences, the endosphere communities of these two plants were strikingly similar. While we did find an effect of plant species on the endosphere community structures (Table 3, Figure 6C), the plants shared a core microbiome, dominated by *Burkholderiales, Actinomycetales* and *Rhizobiales*, across plants in the three arcto-alpine climatic regions. Of these, *Actinomycetales* and *Burkholderiales* have been reported as components of the core root microbiome of, e.g., *A. thaliana* (Schlaeppi et al., 2014). *Rhizobiales* are known plant symbionts with nitrogen fixing abilities, while *Burkholderiales* are well known for their biodegradative capacities and antagonistic properties toward multiple soil-borne fungal pathogens (Benítez and McSpadden Gardener, 2009; Chebotar et al., 2015). In our study, the core microbiome OTUs representing *Burkholderiales*, especially *Comamonadaceae* and *Oxalobacteraceae*, were relatively more abundant in *O. digyna*. We have repeatedly isolated bacteria from *O. digyna* vegetative tissues with very high sequence homologies to the above core OTUs ((Nissinen et al., 2012); unpublished). Further, we have isolated or detected (in clone libraries) bacteria in *O. digyna* seeds with 100% (16S rRNA gene based) identity to six of the core OTUs (OTUs 2 and 16 representing *Rhizobiales*, OTUs 8, 13, and 35 (*Burkholderiales*) and OTU 15 (*Actinomycetales*) (unpublished data). Core OTUs related to similar strains from seeds were highly enriched (Figure 7) in the endosphere or rhizosphere soils. Part of these core organisms could thus be seed-transmitted and colonize the rhizo- and endosphere of developing seedlings, as previously described by Puente et al. (2009) in desert cacti. This indicates the potential importance of such seed-transmitted endophytes in pioneer plants. Horizontal transmission of a set of endophytes has also been observed by Hardoim et al. (2012) and Johnston-Monje and Raizada (2011).

In addition, these core OTUs were among the primary drivers of region, compartment or host plant species differences among the bacterial communities. The higher relative abundances of *Clostridia* in Ny-Ålesund and *Rhizobia* in Mayrhofen in the endosphere communities is one such example, as discussed above.

In summary, we here report that, on the basis of data obtained with two plant species, host plant-specific endophytic communities can be acquired despite a distance of over 3000 km and differences in climate and chemistry between soils. These plant species-specific assemblages are formed from a shared core set of bacteria, most of which are strongly enriched in the endosphere. We surmised that plant-driven selection processes play a role, possibly concomitant with a highly efficient adaptation and fitness of these bacteria in the plant environment. Some of the core OTUs could even be seed-inherited, explaining their tight association with the host plant. Very closely-related endophytic taxa have previously been found to be shared by plants from other cold climates (Nissinen et al., 2012; Carrell and Frank, 2015; Poosakkannu et al., 2015), indicating the ecological tightness of [efficient] establishment of specific bacteria in arcto-alpine plants.

## Author contributions

Study was conceptualized and designed by RN and MK. Field work was performed by MK, RN, and GB. Sample processing was done by MK and RN. Supporting soil analysis was done by MK while library preparation for sequence analysis was done by MK with assistance of AM. Bioinformatics analysis was performed by MK and the data analysis was done by MK and RN. Manuscript draft was prepared by MK, RN, and JE and revisions was done by MK, RN, AM, GB, AS, and JE. Final version for the submission was prepared by MK and RN.

## Nucleotide sequence data

Nucleotide sequence data has been submitted to the ENA database and with accession number PRJEB17695.

## Funding

This research was funded by NWO project 821.01.005 (for JE), Finnish cultural foundation Lapland regional fund (for RN) and by Finnish Academy, project 259180 (for RN)

### Conflict of interest statement

The authors declare that the research was conducted in the absence of any commercial or financial relationships that could be construed as a potential conflict of interest. The reviewer MA and handling Editor declared their shared affiliation, and the handling Editor states that the process nevertheless met the standards of a fair and objective review.
