# Supplementary material for: Plants Assemble Species Specific Bacterial Communities from Common Core Taxa in Three Arcto-Alpine Climate Zones
Source: Front Microbiol. 2017 Jan 24;8:12. doi: 10.3389/fmicb.2017.00012 (PMC5258723; doi:10.3389/fmicb.2017.00012)
Supplement: Supplementary file 1 [file DataSheet1.docx]

**S1 Sampling site coordinates**

Ny-Ålesund, Svalbard

Midtre Lowenbreen N 78.909 E011.998

Red River N 78.934 E011.826

Knudsenheia N 78.936 E011.842

Kilpisjärvi, Finland

Jehkas Old N 69.085 E020.828

Jehkas New N 69.085 E020.823

Saana N 69.052 E020.825

Mayrhofen, Austria

Alps N 47.131 E011.900

Cliff N 47.125 E011.898
